# RNaseH-mediated simultaneous piggyback knockdown of multiple genes in adult zebrafish

**DOI:** 10.1038/s41598-020-76655-5

**Published:** 2020-11-19

**Authors:** Revathi Raman, Mia Ryon, Pudur Jagadeeswaran

**Affiliations:** Department of Biological Sciences, University of North Texas, 1511 West Sycamore Street, Denton, TX 76203 USA

**Keywords:** Biological techniques, Genetics

## Abstract

We recently developed a piggyback knockdown method that was used to knockdown genes in adult zebrafish. In this method, a vivo morpholino (VMO) piggybacks an antisense deoxyoligonucleotide (dO) into the somatic cells and reduces the cognate mRNA levels. In this paper, we tested whether we can piggyback more than one dO with one VMO. We designed various hybrids that had more than one dO that could be piggybacked with one VMO. We chose *f7*, *f8,* and *αIIb* genes and tested their knockdown by the appropriate assays. The knockdown with piggybacking either two or three dOs by one VMO yielded > 85% knockdown efficiency. We also performed knockdown of *argonautes* and *rnaseh* separately along with *f7*. We found the knockdown of *f7* occurs when knockdown of *argonautes* happens and not when *rnaseh* knockdown was performed, suggesting that RNaseH is involved in mRNA degradation. In conclusion, we developed a method where we could knockdown three genes at one time, and by increasing the concentration of VMO by twofold, we could knockdown six genes simultaneously. These multiple gene knockdowns will not only increase the efficiency of the method in whole genome-wide knockdowns but will also be useful to study multifactorial disorders.

## Introduction

Genetic screens have provided information on various physiological pathways with the use of model organisms such as *Drosophila melanogaster*, *C. elegans*, and *Danio rerio* (zebrafish)^1–3^. Ethyl nitrosourea mutagenesis followed by identification of mutants and linkage mapping/positional cloning in a given biochemical/physiological pathway led to the discovery of novel factors^4^. Knockout methods, including the ones involving CRISPR-Cas9, also assisted in understanding the functions of genes^5^. Similarly, knockdown technologies such as siRNA and shRNA have facilitated studies in not only cell culture systems but in whole animals^6–8^. Morpholino knockdown methods that were developed and used in organisms such as zebrafish revolutionized the identification of novel genes/factors^9^. Morpholinos have been modified at the 3′-end to include an octa-guanidine dendrimer moiety such that the morpholino enters the cell. These modified morpholinos are called vivo morpholinos (VMO)^10^. This technology has resulted in knockdown of genes in mice, and it has even led to FDA approved clinical trials to treat Duchene Muscular Dystrophy^11^.

**Figure 1:**
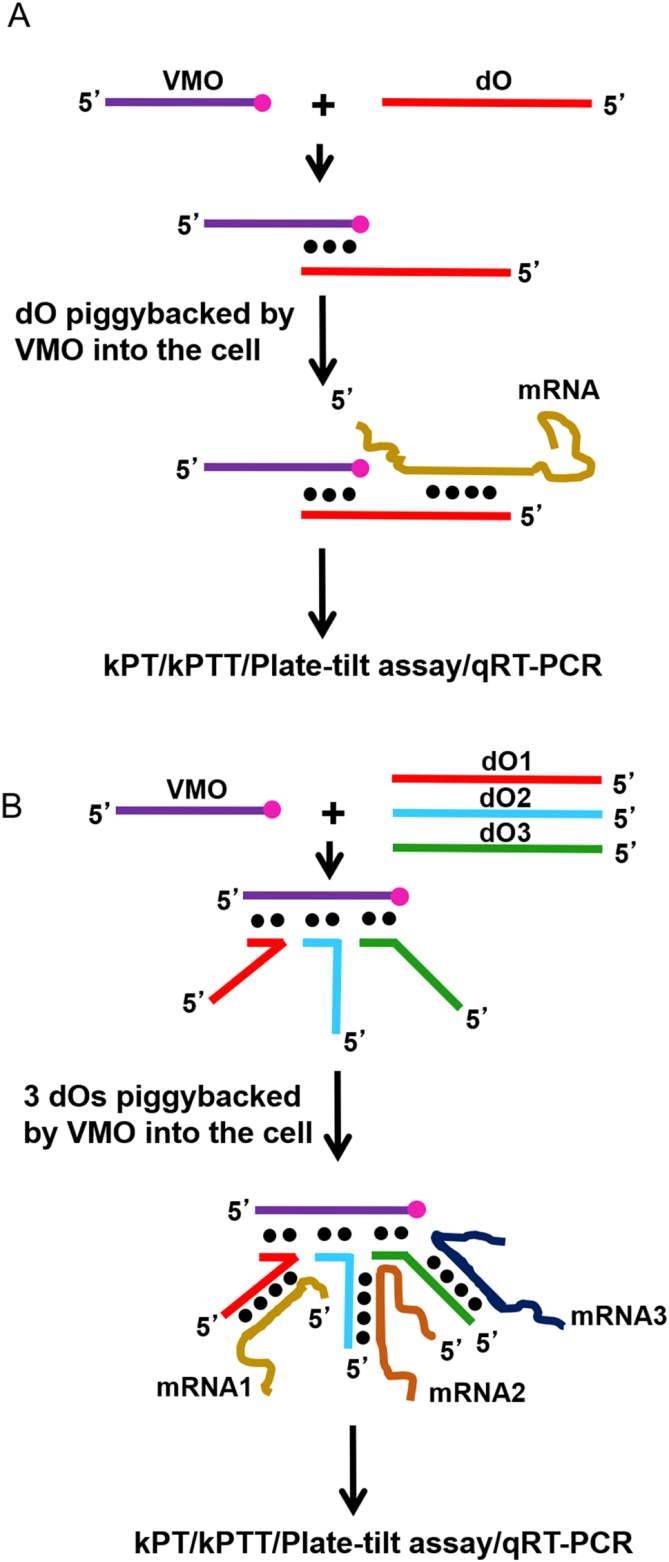
Schematic diagram of the piggyback knockdown. (**A**) The hybrid formation between dO (antisense deoxyoligonucleotide), VMO (vivo morpholino), and mRNA are shown. Black circles indicate base pairing, and pink circle represents octa guanidine dendrimer. The top arrow leads to the formation of the hybrid between VMO and dO in vitro. The middle arrow points to the formation of the hybrid between mRNA and VMO/dO hybrid in vivo after intravenous injections*.* The bottom arrow leads to assays performed 48 h post-injection. (**B**) Simultaneous knockdown of three genes. Note the hybrid formation between three antisense deoxyoligonucleotides, dO1, dO2, dO3, and one VMO at three locations. Also note hybrid formation between three antisense deoxyoligonucleotides, dO1, dO2, dO3, and three mRNAs, mRNA1, mRNA2, and mRNA3. Black circles indicate base pairing, and pink circle represents octa guanidine dendrimer. The top arrow leads to the formation of the hybrid between VMO and 3 dOs in vitro. The middle arrow points to the formation of the hybrid between 3 mRNAs and VMO/3 dOs-hybrid in vivo after intravenous injections*.* The bottom arrow leads to assays performed 48 h post-injection.

We adopted the VMO technology to knockdown genes in both adult and larval zebrafish^12^. However, this VMO technology is prohibitively expensive for large-scale genome-wide knockdowns. Therefore, we developed an inexpensive piggyback knockdown method to study gene function^13^. In this approach, a standard antisense deoxyoligonucleotide (dO) is piggybacked into the cell by a VMO. Here the dO pairs with a VMO using its 3′-end and hybridizes to the mRNA on its 5′-end (Fig. 1). We do not know the mechanism of the piggyback knockdown. Moreover, at present, the efficiency of knockdown is limited to one gene at a time. Thus, for large-scale genome-wide knockdowns, the current method is not conducive.

In this paper, we not only improved the efficiency of the piggyback method by three-fold but also established the knockdown is accomplished by RNaseH-mediated mRNA degradation. We synthesized coagulation factor VII (*f*7), coagulation factor 8 (*f*8)*,* and platelet integrin (*αIIb*) antisense dOs and hybridized all these three antisense dOs with one VMO and simultaneously knocked down three genes (Fig. 1). We found a reduction in clotting and thrombocyte aggregation in the functional evaluation assays. We also found the efficiency of knockdown was greater than 85% for each of these genes as established by quantitative Real-Time PCR (qRT-PCR). Also, we established that knocking down four genes simultaneously is difficult by this method because the five base-pair hybridizations that are required for accommodating four antisense dOs did not result in the knockdown of these genes. However, we found that by doubling the concentration of VMO we could piggyback six dOs and knockdown six genes simultaneously. We also found that the degradation was reduced by knockdown of RNaseH. Thus, this simultaneous multigene piggyback knockdown method should be useful in whole genome-wide knockdown screens for any physiological pathway where the VMO delivery is feasible. Moreover, the ability to simultaneously knockdown multiple genes could be exploited to study multifactorial effects on a given phenotype.

## Results

### Optimization of VMO and dO hybrids

**Figure 2:**
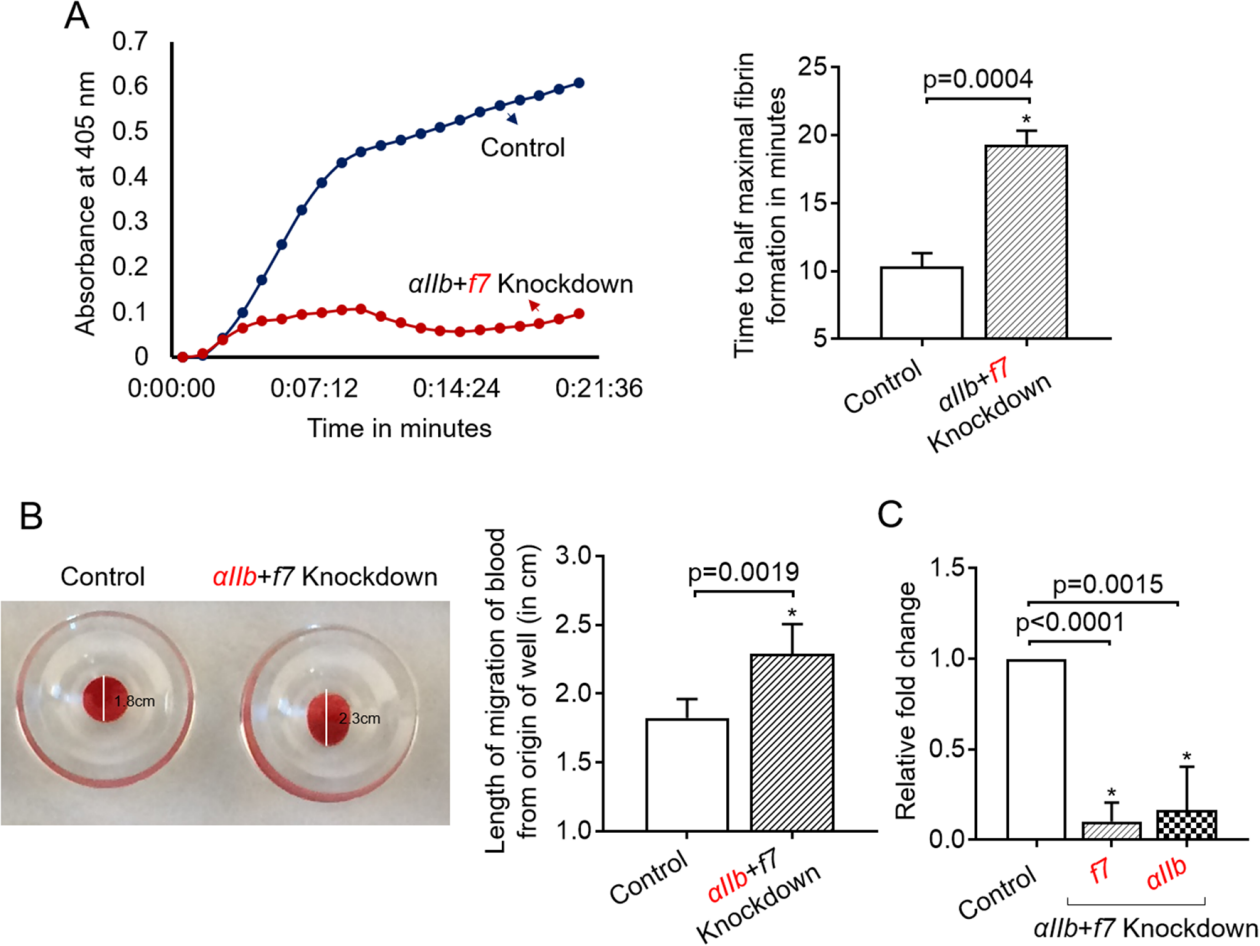
Assays demonstrating the simultaneous knockdown of *αIIb* and *f7* (*αIIb* + *f7*) genes. (**A**) Representative kPT assay curves of fibrin formation (left panel) that show the prolonged initiation of clotting in *αIIb* + *f7* knockdown. The rate curves were generated in the presence of thromboplastin. Time in minutes was plotted against absorbance at 405 nm. The blue and red curves correspond to control and *αIIb* + *f7* knockdown, respectively. kPT curves were generated for control and *αIIb* + *f7* knockdown fish, and from these curves, time taken for half-maximal fibrin formation was plotted (right panel). The time taken for half-maximal fibrin formation from control fishes was compared to *αIIb* + *f7* knockdown fishes by one-way ANOVA; error bars represent standard deviation; (**B**) Collagen-mediated aggregation of whole blood from control and *αIIb* + *f7* knockdown zebrafish. The photograph (left panel) shows the migration of blood from the top of the well (indicated by a vertical white line, the length of migration of blood from the origin of the well is shown in cm) in control and *αIIb* + *f7* knockdown zebrafish when collagen was used as an agonist. The length of migration of blood (right panel) from the origin of the well from control was compared to *αIIb* + *f7* knockdown fish by one-way ANOVA; error bars represent standard deviation; (**C**) Quantitative Real-Time PCRs showing the efficiency of knockdown; the relative fold change in gene expression of *f7* and *αIIb* is shown. For all experiments in (**A**–**C**) panels, p-value < 0.05 was considered significant, and six fishes were used in control and knockdown experiments (N = 6). The functional assay was performed for the knockdown of only one gene shown in red font after simultaneous knockdown of both *αIIb* and *f7*.

**Figure 3:**
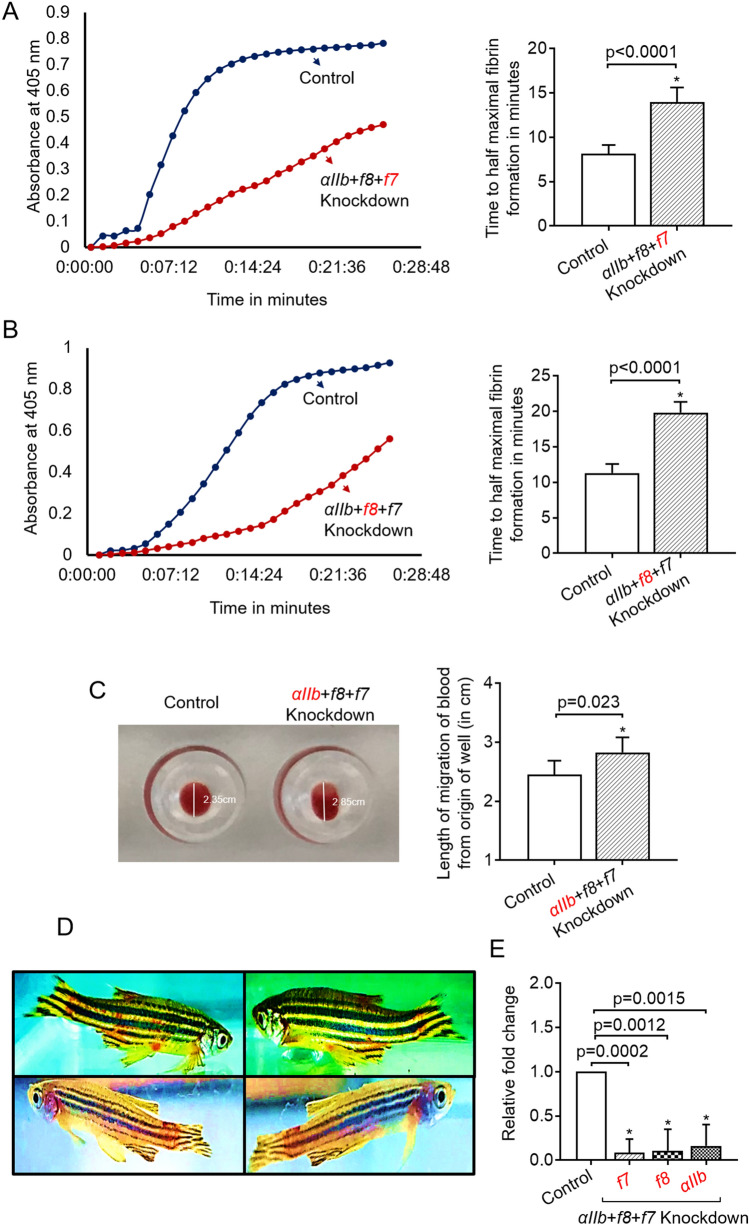
Assays demonstrating the knockdown of *αIIb, f8*, and *f7* (*αIIb* + *f8* + *f7*) genes. (**A**) Representative kPT assay curves of fibrin formation that shows the prolonged initiation of clotting in *αIIb* + *f8* + *f7* knockdown (left panel). The rate curves were generated in the presence of thromboplastin. Time in minutes was plotted against absorbance at 405 nm. The blue and red curves correspond to control and *αIIb* + *f8* + *f7* knockdown, respectively. kPT curves were generated from control and *αIIb* + *f8* + *f7* knockdown fish, and from these curves, time taken for half-maximal fibrin formation was plotted (right panel). The time taken for half-maximal fibrin formation from control fishes was compared to *αIIb* + *f8* + *f7* knockdown fishes by one-way ANOVA; error bars represent standard deviation; (**B**) Representative kPTT assay curves of fibrin formation that shows the prolonged initiation of clotting in *αIIb* + *f8* + *f7* knockdown (left panel). The rate curves were generated in the presence of ACTIN. Time in minutes was plotted against absorbance at 405 nm. The blue and red curves correspond to control and *αIIb* + *f8* + *f7* knockdown, respectively. kPTT curves were generated from control and *αIIb* + *f8* + *f7* knockdown fish, and from these curves, time taken for half-maximal fibrin formation was plotted (right panel). The time taken for half-maximal fibrin formation from control fishes was compared to *αIIb* + *f8* + *f7* knockdown fishes by one-way ANOVA; error bars represent standard deviation; (**C**) Collagen-mediated aggregation of whole blood from control and *αIIb* + *f8* + *f7* knockdown zebrafish. The photograph (left panel) shows the migration of blood from the top of the well in control and *αIIb* + *f8* + *f7* knockdown zebrafish when collagen was used as an agonist (indicated by a vertical white line, the length of migration of blood from the origin of the well is shown in cm). The length of migration of blood (right panel) from the origin of the well from control was compared to *αIIb* + *f8* + *f7* knockdown fish by one-way ANOVA; error bars represent standard deviation; (**D**) Zebrafish showing bleeding after simultaneous knockdown of three genes *αIIb* + *f8* + *f7* (4 out of 18 fishes from the above three experiments, (**A**–**C**) developed bleeding); (**E**) Quantitative Real-Time PCRs showing the efficiency of knockdown. Relative fold change in gene expression of *f7*, *f8*, and *αIIb* is shown. For all experiments in (**A**–**C**), p-value < 0.05 was considered significant, and 6 fishes were used in control and knockdown experiments (N = 6). The functional assay was performed for the knockdown of only one gene shown in red font after the simultaneous knockdown of *αIIb*, *f8*, and *f7*.

We have previously reported a piggyback knockdown where an *αIIb* gene-specific antisense dO is piggybacked by a VMO that resulted in knockdown of *αIIb*. However, if this method were to be used for the whole genome-knockdown of 25,000 genes, one gene at a time, it would be a tremendous undertaking. Thus, we hypothesized that if more than one dO can be piggybacked by the VMO, then multiple genes could be knocked down in a single zebrafish and if the knockdown does not affect the phenotype we can rule out multiple genes affecting that phenotype. If we observe any effect on the phenotype, then by repeating the injections of each of these dOs separately, we can identify the correct gene involved in the above phenotype. To test this hypothesis, we designed hybrids that had more than one dO that could be piggybacked by one VMO. In this design, since we have only 25 bases in the VMO, we first designed two dOs, each 35 nt in length, one for *f7* gene, and one for *αIIb* gene. The first 10 bases (at 3′-end) of *f7* and *αIIb* dOs hybridize with VMO at two different locations from 5′-end to 3′-end of the VMO, respectively. The rest of the 25 bases of these two dOs were antisense to *f7* and *αIIb* mRNAs. We injected the above hybrids into the fish, and after 48 h, the blood was collected from the fish, and the plasma and blood were assayed for *f7* and *αIIb* activity by kPT, and plate-tilt assay, respectively. We found kPT was prolonged, and the length of blood flow from the origin was longer than the control suggesting the knockdown of *f7* and *αIIb*, respectively (Fig. 2). We also performed qRT-PCRs on the brown mass RNA to check the efficiency of knockdown for these two genes. The results showed that these two genes were knocked down by more than 75% (Fig. 2). Then we checked whether we could piggyback three dOs simultaneously with one VMO. We designed *f7*, *f8,* and *αIIb* antisense dOs for targeting the respective mRNAs. This time we adjusted the length of hybridizing base pairs to VMO to be 7 such that each of these dOs is arranged sequentially from the 5′-end of VMO. This arrangement accommodates the base pairing of all the three different dOs. We then performed kPT, kPTT, and plate-tilt assay for assaying *f7*, *f8*, and *αIIb*, respectively. The results showed prolongation in kPT, and kPTT curves, and plate-tilt assay showed greater length of migration (Fig. 3) of blood suggesting the knockdowns worked. We also used qRT-PCR to check the efficiency of knockdown for these three genes. The results showed that all three genes were knocked down by more than 75% (Fig. 3). Interestingly as expected, we also noted bleeding spots in 22% of the injected fish (Fig. 3). We then tested whether we can piggyback antisense dOs corresponding to *f7*, *f8*, and *αIIb* genes by reducing the hybridizing bases to 5 instead of 7 to accommodate four dOs. We performed qRT-PCRs as mentioned above for these genes, and the results showed no difference between the control and experimental groups (data not shown).

### Mechanism of knockdown

**Figure 4:**
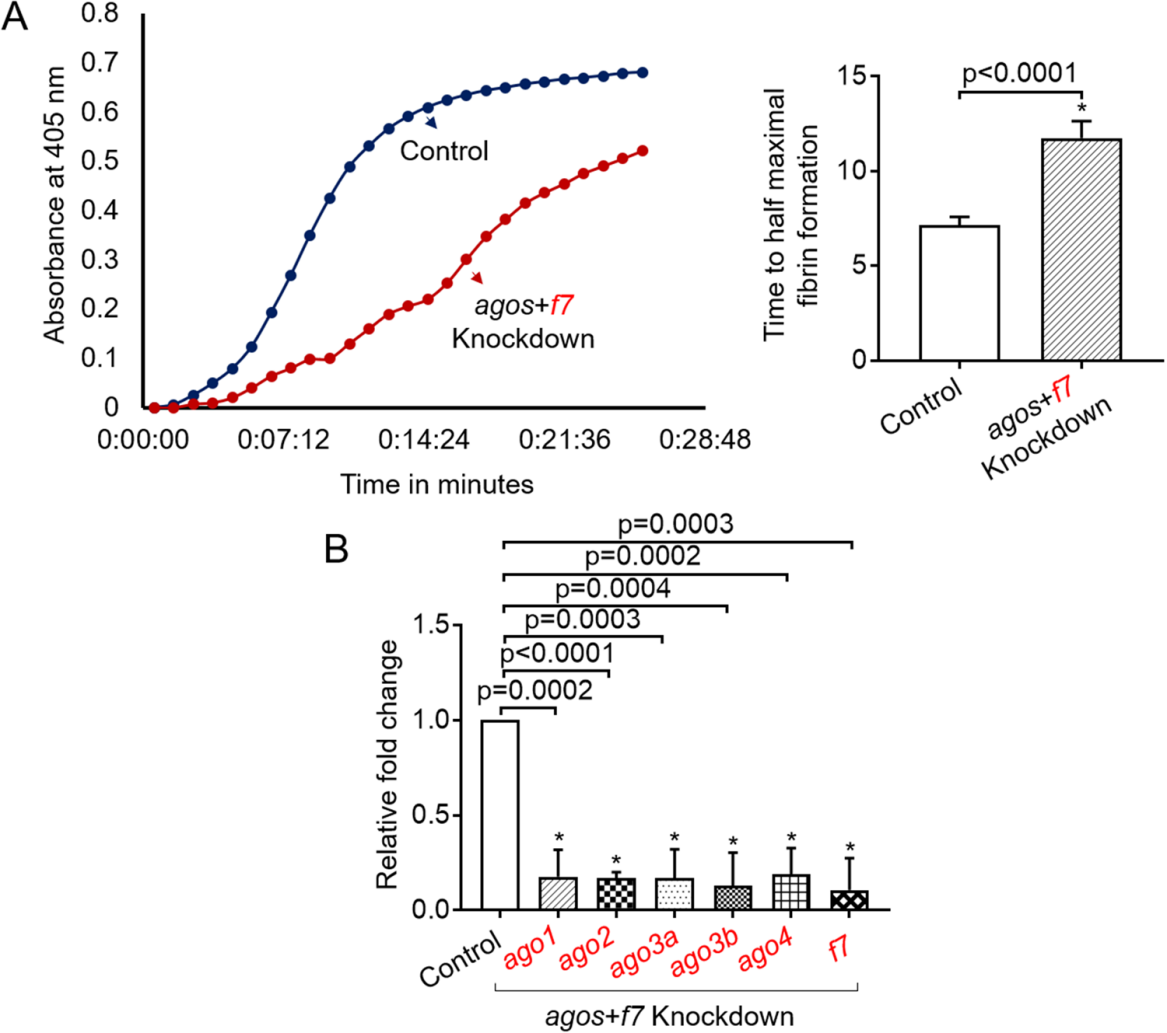
Assays demonstrating the knockdown of *argonautes*, *ago1*, *ago2*, *ago3a*, *ago3b*, *ago4,* and *f7* (*agos* + *f7*). (**A**) Representative kPT assay curves of fibrin formation that shows the prolonged initiation of clotting in *ago* + *f7* knockdown (left panel). The rate curves were generated in the presence of thromboplastin. Time in minutes was plotted against absorbance at 405 nm. The blue and red curves correspond to control and *agos* + *f7* knockdown, respectively. kPT curves generated from control and *agos* + *f7* knockdown fish, and from these curves, time taken for half-maximal fibrin formation was plotted (right panel). The time taken for half-maximal fibrin formation from control fishes was compared to *agos* + *f7* knockdown fishes by one-way ANOVA; error bars represent standard deviation; (**B**) Quantitative Real-Time PCRs showing the efficiency of knockdown. Relative fold change in gene expression of *ago1*, *ago2*, *ago3a*, *ago3b*, *ago4*, and *f7* is shown. For all experiments in (**A**,**B**), p-value < 0.05 was considered significant, and 6 fishes were used in control and knockdown (N = 6). The functional assay was performed for the knockdown of only one gene shown in red font after the simultaneous knockdown of *agos* and *f7*.

Since the piggybacked oligonucleotide knockdown probably involves mRNA degradation and the mechanism of this degradation is not known, we explored the mechanism of the piggyback method of RNA silencing. Although RISC complex silencing by RNA-RNA hybrid formation is well known, there are reports that DNA-mediated RNA degradation is possible^14^. However, since in zebrafish, we do not know whether such mechanism exists, we first explored whether argonautes are involved in the degradation of mRNA in the piggyback method. We tested this by checking whether the knockdown of *argonaute* genes affects the degradation of *f7* mRNA in its piggyback knockdown. Since there are 5 *argonaute* genes in zebrafish and one functional *f7* gene, we wanted to knockdown all six genes simultaneously. Therefore, we increased the concentration of VMO twofold such that we could hybridize all six dOs as two different sets, each set having a combination of three dOs (first set for *ago1*, *ago2*, and *ago4* and the second for *ago3a*, *ago3b*, and *f7*). The hybrid carrying six antisense dOs was injected into zebrafish, and after 48 h, we performed kPT assay. The results showed a prolongation in kPT, suggesting that the lack of argonautes did not inhibit the degradation of *f7* mRNA, and in fact, the knockdown was occurring (Fig. 4). We tested for the knockdown of these six genes by qRT-PCR and found that knockdown of all six genes was greater than 75% (Fig. 4).

**Figure 5:**
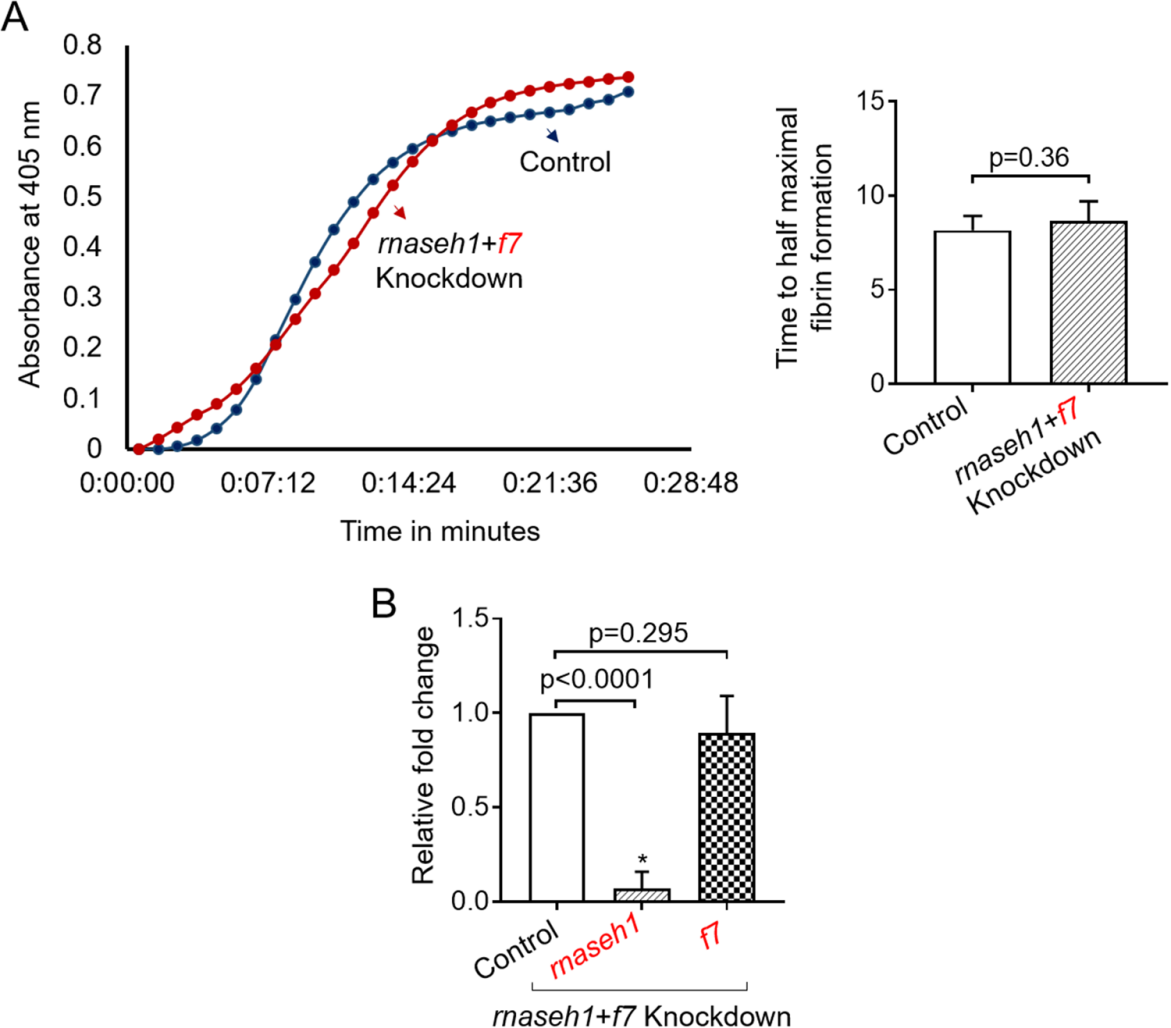
Assays demonstrating the knockdown of *rnaseh1* and *f7* (*rnaseh1* + *f7*). (**A**) Representative kPT assay curves of fibrin formation showing similar initiation and progression of clotting in *rnaseh1* + *f7* knockdown compared to that of control (left panel). The rate curves were generated in the presence of thromboplastin. Time in minutes was plotted against absorbance at 405 nm. The blue and red curves correspond to control and *rnaseh1* + *f7* knockdown, respectively. kPT curves were generated from control and *rnaseh1* + *f7* knockdown fish, and from these curves, time taken for half-maximal fibrin formation was plotted (right panel). The time taken for half-maximal fibrin formation from control fishes was compared to *rnaseh1* + *f7* knockdown fishes by one-way ANOVA; error bars represent standard deviation; (**B**) Quantitative Real-Time PCRs showing the efficiency of knockdown. Relative fold change in gene expression of *rnaseh1* and *f7* is shown. For all experiments in (**A**,**B**), p-value < 0.05 was considered significant, and 6 fishes were used in control and knockdown experiments (N = 6). The functional assay was performed for the knockdown of only one gene shown in red font after the simultaneous knockdown of *rnaseh1* and *f7*.

Since it is well known that RNaseH degrades RNA of the RNA:DNA hybrid and phosphorothioate antisense oligodeoxynucleotides have been used in degrading mRNA in Xenopus oocytes^15,16^, we wanted to examine whether an RNaseH-mediated degradation occurs in the piggyback knockdown method. Since there is one *rnaseh1* gene in zebrafish, we performed knockdown by using two antisense dOs for *rnaseh1* along with antisense dO for *f7*. The results showed that the *rnaseh1* knockdown resulted in inhibition of *f7* mRNA degradation, suggesting the pathway of inhibition is via RNaseH (Fig. 5).

## Discussion

In this paper, we have developed a method to knockdown multiple genes simultaneously in adult zebrafish by intravenous injections of knockdown hybrids. Thus, for 25,000 gene-knockdowns with a combination of six antisense dOs, it should only take 4000 single fish injections to screen the entire genome to identify genes involved in a given physiological pathway. Therefore, limiting 100 injections per person per day and 100 phenotypic tests per day, it should only take 40 days to perform whole-genome knockdowns. Even if we use six fish for knockdown of each gene to provide statistically meaningful results, a single individual can perform the whole-genome knockdowns in less than a year. Once primary screening with the six gene-knockdowns are done several sets of six genes can be eliminated. Then in the secondary screening, the individual gene knockdowns in sets that are positive can be performed to identify the genes involved in the pathway. Similarly, a tertiary screen on the individual genes can be performed for confirmation.

In our analysis, we have used genes expressed in the liver, endothelium, and thrombocytes. Since brown mass consists of all the three types of cells, one RNA preparation was sufficient. Our data revealed all the genes expressed in the above tissues/cells are undergoing knockdown. Our success in knockdowns is probably because intravascular injections may facilitate piggyback VMO to target highly vascularized tissues. Since we assay 48 h post-injection, long half-life proteins may be difficult to knockdown. The knockdown of these long half-life candidates would require repeated injections of knockdown reagents.

Our technology also depends on successful intravenous injections and the quantity of a hybrid solution that enters circulation. Thus, for each set of six genes, the accuracy of the delivery of the knockdown hybrids is required. Due to these variables for each set of six genes, at least 6 fish injections are required. One future application of this technology would be studying the combinatorial effects of multiple gene knockdowns. Although this method has an inherent advantage to study multifactorial genetics, the combinations of six genes in a whole-genome setting are astronomical. However, if candidate genes are available and their combinations have to be studied, this technology would be an ideal platform. Furthermore, for one gene, multiple dOs could be used to obtain almost 100% knockdown. Also, for fatal disorders where the gene products must be silenced, this technology should be useful.

Despite the fact that whole genome knockdowns could identify the genes that contribute to a given pathway, caution must be exercised because mutations in the same genes may not yield the phenotypes due to genetic compensation^17,18^. This compensatory effect could also be there even in knockdowns. Thus, gene knockdowns may not detect such genes. However, when we suspect other orthologous genes could play the role, we may target these genes by our approach of multiple knockdowns. Despite the above issue, genome-wide screens will establish a database of genes that might be affected and could be eliminated later when mutants are generated or available. In addition to the above genetic compensation issue, there are multiple points to be considered in the whole-genome knockdowns. First, the design of antisense oligonucleotides is critical. The oligonucleotides described in this paper are similar to the design of the hybridization primers. The available primer design programs such as Primer3 will address the formation of the hairpin and optimal GC content. Nevertheless, off-target issues exist in the knockdown technologies that depend on base pairing, which could be minimized by using p53 antisense oligonucleotides, because p53 knockdowns are supposed to minimize the off-target effects^19–21^. Second, the dosage optimization of the hybrids we are injecting requires attention. Here, we inject the highest dose possible for the hybrids with almost 20 μM final concentration in the 50 μl blood volume. At such concentrations, most proteins (low abundant to high abundant) so far tested appear to work. Interestingly, it has been shown that acetylcholine receptor channel, an abundantly expressed protein was knocked down efficiently^22^. However, for some high abundant mRNAs, it may not knockdown completely. In such cases, repeated injections are necessary, and thus initial screening may not detect these as knockdowns. This is one of the limitations of this technology. Third, we need to consider whether the hybrid injected will reach all the tissues. As mentioned above, highly vascularized tissues will certainly be exposed, and thus the genes will be knocked down in these tissues. It has been shown in mice that knockdown occurs in most tissues^10^. However, we do not know whether the hybrid crosses the blood–brain barrier. Thus, targeting such tissues may require intracerebroventricular injections, as has been done by laboratories interested in knockdown in brain tissue^23,24^. Despite all the limitations, the method developed here is good for initial genome-wide screening to discover novel genes expressed in many tissues, which can later be confirmed with knockout technologies. In summary, we developed a multigene piggyback knockdown method that should be useful in whole-genome knockdown screens as well as in accomplishing almost 100% knockdown of a specific gene. It may have therapeutic applications, especially since VMO has been approved by FDA.

## Methods

### Zebrafish husbandry

Adult wild-type zebrafish (AB strain) were obtained from Ekkwill farms via the Fish N Chirps Pet Center, Denton TX, and maintained at 28 °C in the circulating system of deionized water that was supplemented with Instant Ocean. The fish were kept in 14 h of light and 10 h of dark cycle and fed with brine shrimp and flakes.

### Piggyback reagents and injection

From the predicted protein-coding sequence of *f7, f8, αIIb, ago1, ago2, ago3a, ago3b, ago4, and rnaseh1*, the corresponding coding cDNA sequence was chosen. Then approximately in the middle of the coding sequence, 25 nucleotide-long antisense sequence was selected (Table 1). To this sequence, a 10, 7, and 5 nucleotide-long sequence was added at its 3′-end, called as antisense dO. The above 3′-end sequence base pairs with a VMO (Table 1). The oligonucleotides were purchased from Invitrogen, Carlsbad, CA. VMO (5′-CCTCTTACCTCAGTTACAATTTATA-3′) was purchased from Gene-Tools LLC, Philomath, OR. The hybrid was prepared with final concentrations of 0.25 mM VMO and 0.25 mM of each antisense dO containing oligo-hybridization buffer 50 mM NaCl, 1 mM Tris–HCl (pH 8.0), and 0.1 mM EDTA (pH 8.0). The above mixture was heated to 90 °C and slowly cooled to room temperature using the Takara PCR Thermal Cycler.

**Table 1:** Knockdown primers used in gene knockdown experiments

Gene name	KD primer (5′ to 3′)	VMO (5′ to 3′)
*αIIb*	CTCGATAATCAGCAGCGCTGGATTC**TATAAATTGT**	CCTCTTACCTCAGTT**ACAATTTATA**
*f7*	CAGTGACCTTTAGGACATTCAGATC**AGGTAAGAGG**	**CCTCTTACCT**CAGTTACAATTTATA
*αIIb*	CTCGATAATCAGCAGCGCTGGATTC**TATAAAT**	CCTCTTACCTCAGTTACA**ATTTATA**
*f8*	CCGTAGTAATCAGACGCTGTTATTT**TAACTGA**	CCTCTTACC**TCAGTTA**CAATTTATA
*f7*	CAGTGACCTTTAGGACATTCAGATC**TAAGAGG**	**CCTCTTA**CCTCAGTTACAATTTATA
*αIIb*	CTCGATAATCAGCAGCGCTGGATTC**TATAA**	CCTCTTACCTCAGTTACAAT**TTATA**
*f8*	CCGTAGTAATCAGACGCTGTTATTT**TGTAA**	CCTCTTACCTCAG**TTACA**ATTTATA
*f7*	CAGTGACCTTTAGGACATTCAGATC**GAGGT**	CCTCTT**ACCTC**AGTTACAATTTATA
*ago1*	AAGGATCCAGATTAAAGTTAGCGTT**TATAAAT**	CCTCTTACCTCAGTTACA**ATTTATA**
*ago2*	TGTACTTATCCTTGAAGTACTGGGC**TAACTGA**	CCTCTTACC**TCAGTTA**CAATTTATA
*ago4*	ACAATGCCAAACTCTTTCAGATAAG**TAAGAGG**	**CCTCTTA**CCTCAGTTACAATTTATA
*ago3a*	ACTGAGCTACTGTCCGTTCTACAGT**TATAAAT**	CCTCTTACCTCAGTTACA**ATTTATA**
*ago3b*	AAGGTAGGTATGTGTGTTTCTGCTC**TAACTGA**	CCTCTTACC**TCAGTTA**CAATTTATA
*rnaseh1*	ACCTTTTTAAAATTCATTTCCTTGG**TATAAAT** GCAAACTTCTTAAAACTTGCAGAAG**TAACTGA**	CCTCTTACCTCAGTTACA**ATTTATA** CCTCTTACC**TCAGTTA**CAATTTATA

Note the sequences from Knockdown (KD) primer that base pairs with VMO sequences are shown in bold font.

Adult zebrafish were injected intravenously with 5 µl of either the above hybrid or 1X PBS. For these injections, 5 µl of the hybrid or 1X PBS was pipetted onto a parafilm. A 27G11/4 needle was placed onto a 1 ml syringe. The 5 µl on the parafilm was gently sucked into the needle portion of the syringe. To assure that it does not get sucked beyond the needle, we eject the 5 µl back onto the parafilm and suck it back again gently. A zebrafish was placed on a dry Kimwipe, cover its head with the Kimwipe so it does not jump or flip, and its body was wiped dry. The ventral fin was slightly pushed onto the side such that we can visualize the origin of the ventral fin. In line with the origin of the ventral fin, towards the dorsal fin, we chose a spot on the second dark stripe from the ventral side underneath where the inferior vena cava resides. The needle was then kept at that spot at approximately 5- to 10-degree angle to the body and then gently pushed, so the needle enters into the vessel. The syringe piston was immediately pushed gently to inject the contents. The success of the injection is usually evident when we see a blood spot at the site of injection. The fish was then returned to the tank. Video S1 shows the injection of 5 µl of 1X PBS.

### Blood collection

On a clean paper towel, zebrafish was placed on its lateral side, head covered with a wet Kimwipe, and the skin surface was gently wiped with Kimwipe. After making a lateral incision between the ventral and the dorsal fins and in between second and fourth black stripes from the dorsal end, blood was collected by using a pipette tip and was used in further experiments^25,26^. The above procedure was approved by the Institutional Animal Care and Use Committee of the University of North Texas, and animal experiments were performed in compliance with the institutional guidelines.

### RNA isolation

Total RNA was isolated using TRI Reagent (Sigma-Aldrich, St. Louis, MO), 1-bromo-3-chloropropane (BCP), and isopropanol. The brown mass consisting of liver and spleen was collected from anesthetized adult zebrafish after laparotomy. TRI reagent was added according to the manufacturer’s recommendations. This sample was then gently homogenized using Branson Sonifier 250, and to this homogenate, BCP was added. After centrifugation, the aqueous phase was precipitated by isopropanol. The dry pellet was then suspended in nuclease-free water. The total RNA concentration was measured using NanoDrop.

### Quantitative RT-PCR (qRT-PCR)

The forward primer (FP) and reverse primer (RP) from coding sequences of *f7, f8, αIIb, ago1, ago2, ago3a, ago3b, ago4, and rnaseh1* genes were purchased from Invitrogen, Carlsbad, CA (Table 2). 1 µg of total RNA was converted to cDNA at 42 °C for 30 min using qScript cDNA SuperMix (Quantabio, Beverly, MA) and qRT-PCR was performed using PerfeCTa SYBR Green SuperMix (Quantabio, Beverly, MA). The result was analyzed to determine ΔC_T_ values from which relative fold change was measured, and the graph was plotted.

**Table 2:** Forward and reverse primers used in qRT-PCRs

Gene name	qRT-PCR primers (5′ to 3′)
*αIIb*	**FP:** GGAGGTACCTTTGTGGTGGA **RP:** AGGACGGTTACGCTTGAAAA
*f8*	**FP:** TGTCAGCAATGATGGACACA **RP:** CGGATGAAACGAGTGAAGAG
*f7*	**FP:** TGCTTGGAAAAGCTCAAGGT **RP:** CAGGGTGTGTGAACATCTGA
*ago1*	**FP:** CGATACCATCTGGTGGACAA **RP:** GGCAAAATACATGGTCCTCA
*ago2*	**FP:** GCTGTGCCACACCTATGTTC **RP:** GATGTGTGACTGCCTTCAGC
*ago4*	**FP:** GACTCTTCTGCTCCGACAAA **RP:** CATGGCTGCACAGGTAGAAG
*ago3a*	**FP:** GTGGAAACATTCCTGCTGGT **RP:** GCAGTGAAGCAGTTGTCGTC
*ago3b*	**FP:** CTGTGCAGTCATGCTGGAAT **RP:** GCACAGCTGGTACGTCAAGA
*rnaseh1*	**FP:** TGGTGGGGTCATAATCAACA **RP:** ACAGTCTGTCTGCCTCCTCA

### kPT and kPTT assays

2 µl of blood was mixed with 0.5 µl of 3.8% sodium citrate, and the plasma was obtained from citrated blood by centrifugation at 3000 rpm for 10 min at 4 °C. (2 µl of blood from individual fish yielded 1 µl of plasma). Coagulation assays were performed on 96-well flat-bottomed microtitre plates kept on ice. 20 µl of fibrinogen (10 mg/ml) and 1 µl of zebrafish plasma were mixed in each well. 10 µl of either zebrafish thromboplastin (for kinetic prothrombin time, kPT) or Dade ACTIN (for kinetic partial thromboplastin time, kPTT) was added to each well. Each reaction was brought to 94 µl using PBS and recalcified using 6 µl of 100 mmol/l CaCl_2_. The plate was removed from the ice, wiped thoroughly with Kimwipe, and then shaken for 1 min in the Synergy H1 reader (Biotek). The absorbance representing fibrin formation was recorded at 405 nm every minute for 25 min, and this fibrin formation curve was called kPT/kPTT assay clotting curves. Negative controls (flat curves, not shown) were performed by replacing thromboplastin or ACTIN with PBS^27^.

### Whole blood aggregation assay

Aggregation of thrombocytes in response to collagen (1.9 mg/ml) was assayed by the plate tilt method using whole blood. PBS (7 µl) was placed in Nunc microtitre plates containing conical wells (10 µl capacity) followed by 2 µl of collagen. 1 µl of whole blood was then added from the top such that blood goes through the collagen solution to the bottom of the well. After 5 min, the plate was tilted 45° for 30 s. The photograph of the blood migrating down the sloped well wall was acquired. To normalize different pictures, we sized the images such that the diameter of the well was the same in all pictures. The lack of or slower mobility of blood down the slope indicated a positive aggregation in response to agonists and vice versa^25^. To measure the length of blood migration from the top of the well down the wall, we placed a line from the top of the well to the end of migrated blood in the image, the images were printed and the size of the line was assessed using a ruler. All the above procedures use reagents that do not pose any safety hazards and is approved by the Institutional Biosafety Committee.

### Statistical analysis

Statistical analysis was performed using GraphPad Prism7. Groups were compared by one-way ANOVA on ranked data; p < 0.05 was considered statistically significant. Error bars represent standard deviation.

## Supplementary information

Supplementary Video 1.

Supplementary Information 1.
