# Supplementary material for: RNaseH-mediated simultaneous piggyback knockdown of multiple genes in adult zebrafish
Source: Sci Rep. 2020 Nov 19;10:20187. doi: 10.1038/s41598-020-76655-5 (PMC7677540; doi:10.1038/s41598-020-76655-5)
Supplement: Supplementary file 2 — Supplementary Information 1. [file 41598_2020_76655_MOESM2_ESM.docx]

**RNaseH-mediated Simultaneous Piggyback Knockdown of Multiple Genes in Adult Zebrafish**

Revathi Raman, Mia Ryon and Pudur Jagadeeswaran

Department of Biological Sciences, University of North Texas, Denton TX 76203

**Video legend**

Video S1: Intravenous injection of adult zebrafish. Adult zebrafish were injected intravenously with 5 µl of either the piggyback hybrid or 1X PBS. For these injections, 5 µl of the hybrid or 1X PBS was pipetted onto a parafilm and using a 27G11/4 needle 5 µl placed on the parafilm was gently sucked into the needle. A zebrafish that was wiped dry with a Kimwipe was injected on the second dark stripe in line with the origin of the ventral fin, towards the dorsal fin. The success of the injection is usually evident when we see a blood spot at the site of injection. The fish was then returned to the tank.
